# Sexual workplace violence in the health sector in Saudi Arabia: a cross sectional study

**DOI:** 10.1186/s12913-023-10080-y

**Published:** 2023-10-06

**Authors:** Aseel Khaled AlHassan, Reem Tarik AlSaqat, Fahad Saleh Al Sweleh

**Affiliations:** 1 King Khalid Hospital in AlKharj, Ministry of Health, P.O. BOX: 52166, Riyadh, Kingdom of Saudi Arabia; 2 Princess Nourah bint Abdulrahman University, Riyadh, Kingdom of Saudi Arabia; 3 Dental University Hospital, King Saud medical city, King Saud University, Riyadh, Kingdom of Saudi Arabia

**Keywords:** Sexual violence, Survivors, Healthcare workers, Saudi Arabia, Abuse, Violence, Sexual harassment

## Abstract

**Background:**

Sexual workplace violence occurs worldwide with increasing prevalence, causing psychological and physical injuries. However, only few reports from the Kingdom of Saudi Arabia have investigated the most involved health specialty and its association with other factors, such as working and sociodemographic conditions. The aim of this study was to determine the prevalence of workplace sexual violence over 12 months, from May 2018 to May 2019, circumstances related to the event, and consequences for the perpetrator and survivor and to identify associated factors among all healthcare workers (HCWs) in Saudi Arabia.

**Methods:**

This cross-sectional study included all HCWs registered with the Saudi Commission for Health Specialties who worked for > 1 year in the health sector (government or private) in Saudi Arabia until May 2019. A non-probability convenient sampling technique was used. A modified self-administered questionnaire sent via email was utilized to assess workplace violence. Descriptive statistics were used to report percentages and frequencies, while advanced statistics, such as bivariate analysis, were used to determine associations. Multivariate logistic binary regression analysis was used to assess the combined and individual associations between relevant predictors of exposure of HCWs to recent sexual violence at the workplace.

**Results:**

In total, 7,398 (male, 51.3%; female, 48.7%) HCWs were electively enrolled in the study (mean age 40 ± 8.62 years). Most were non-Saudi (60%). Overall, 3.9% were sexual violence survivors. Approximately 60.7%, 51.4%, 48.3%, and 65.9% of female workers, nurses, Saudi natives, and night shift workers (18:00 to 07:00), respectively, were significantly exposed to sexual violence. Furthermore, approximately 54.8% of those with direct physical contact with patients had a higher rate of exposure to sexual harassment (*p* = 0.001).

**Conclusions:**

The prevalence of sexual violence is low but remains a risk to HCWs, especially those working night shifts and having direct physical contact with patients. Thus, more support, specific strategies, and policies are needed to reduce the rate of occurrence, protect HCWs, and prevent such events. The underreporting of cases may be skewing the magnitude of the problem; thus, more education and additional research in Saudi Arabia are needed regarding sexual violence experienced by HCWs.

## Background

The World Health Organization (WHO) defines violence as "the intentional use of physical force or power, threatened or actual, against oneself, another person, or against a group or community, that either results in or has a high likelihood of resulting in injury, death, psychological harm, maldevelopment, or deprivation" [1]. Workplace violence (WPV) could be physical or psychological, including verbal violence, bullying/mobbing, racial harassment, and sexual violence [1]. Although the prevalence of sexual violence is lower than that of other types of violence [2–4], it should not be overlooked, as it impacts the health and quality of life of the harassed persons (hereafter survivors) negatively [5, 6]. According to the WHO, sexual violence is “any unwanted, unreciprocated, and unwelcome behavior of a sexual nature that is offensive to the person involved, and causes that person to feel threatened, humiliated, or embarrassed” [7]. In a systemic review and meta-analysis, Worke et al. [8] reported a prevalence of workplace sexual violence in all Ethiopian workplaces of 22%. In another review of patient violence against healthcare workers (HCWs) in psychiatric inpatient wards, the rate of sexual WPV was 9.5–37.2% [9]. Other reviews have reported varying rates of 0.3% in Taiwan [3], 12% in Ghana [10], and 73% in Turkey [5]. Another systematic review and meta-analysis of sexual WPV inflicted by patients and visitors reported a rate of 14.2% [2]. These variations may be due to different understandings of the meaning of sexual violence in different cultures and the availability of staff per population, noting that the lower the ratio, the heavier the workload, and the less time available for proper communication with the patients [2]. In Saudi Arabia, a conservative Arabic community, sexual harassment is a very sensitive issue. Reported rates of work violence in specific localities around Saudi Arabia ranged from 3% to 76.5% [4, 11–13]. None of these studies focused on sexual WPV and were conducted in certain cities in Saudi Arabia, in hospitals in the same city and same departments, such as the nursing or emergency department. The reluctance of victims to report incidents may be due to the fear of potential repercussions, such as damage to their professional or personal reputation, or the possibility of retaliation from the perpetrator [14]. Sexual attack can result in fear, safety concerns, injury, work leave [15], and diminished work quality [16]. Other effects include psychological disorders, such as anxiety, depression, posttraumatic stress, and/or eating disorders [6]. This low reporting rate can lead to underestimation of the problem and therefore imposes the need for stricter regulations and mechanisms to prevent the attacks and deal with their side effects.

Previous studies have focused on sexual WPV in high-risk environments and specialties in some cities in Saudi Arabia. To our knowledge, none of these studies covered the whole of Saudi Arabia and all specialties. In addition, few studies have focused on the association between WPV and independent risk factors, such as sociodemographic factors, working conditions, and factors from hospital violence reports.

Therefore, this study aimed to determine the prevalence of sexual WPV over a period of 12 months, circumstances related to events, consequences for attackers and survivors, target populations at all healthcare provider facilities in Saudi Arabia, and the most susceptible group of healthcare providers. We also identified the factors associated with WPV in healthcare facilities in Saudi Arabia.

## Methods

### Data collection

An analytical cross-sectional study was conducted between November 4, 2018, and July 1, 2019, among all healthcare providers registered in the Saudi Commission for Health Specialty (SCFHS) and who had been working for more than 1 year in the health sector (government or private) in Saudi Arabia as of May 2019. A non-probability convenient sampling technique was used; the desired sample size was determined based on a maximum variance assumption of 50% that the healthcare workers would report a positive experience of the types of abuse studied. The desired sample size required to detect the true proportion of individuals who had experienced any type of abuse studied with 95% confidence and a margin of error equal to 5% was deemed to be 384. All eligible participants (i.e., physicians, pharmacists, nurses, midwives, health specialists, healthcare technicians, and ambulance personnel) were invited to participate in the study. A total of 304,002 healthcare providers met the eligibility criteria. Students, interns, employees of the administrative department, healthcare providers not registered in the SCFHS, or providers with less than 1 year of work experience were excluded.

Data were collected using a modified self-administered questionnaire developed by the Joint Program on Workplace Violence in the Health Sectors of the WHO, International Labour Organization, International Council of Nurses, and Public Services International [17]. One of the authors (AH) translated the questionnaire into Arabic for staff who were not fluent in English. The questionnaire was then revised by the other two authors (FS and RS) who are both bilingual. Questions that did not apply to Saudi Arabia were omitted.

A pilot test was conducted for reliability and validity by distributing the questionnaire to five physicians, five dentists, five nurses, and five pharmacists, who were both Arabic and English speakers and had clinical experience in validating the Arabic translation to avoid misunderstandings; these practitioners were excluded from the main study.

The questionnaire included questions related to demographic data of the respondents, workplace characteristics, violent events in the previous 12 months, risk factors for WPV, personal opinions, perceptions, attitudes, experiences, and WPV-related recommendations. The questionnaire had a total of 88 questions divided into five sections: personal and workplace data, physical workplace violence, verbal abuse, bullying/mobbing, and sexual harassment.

The questionnaires were e-mailed to the study population by the researchers. To increase the response rate, the researchers sent reminder emails to the participants after 2 weeks.

### Data analysis

Data were entered into SPSS IBM (Version 22). Descriptive statistics (frequency and table) were used to describe the basic features of the data. Continuous variables are expressed as mean and standard deviation (SD), whereas categorical variables are expressed as frequencies and percentages. Multiple response dichotomy analysis was used to describe the items measured with dichotomies (“tick all that apply to you” questions). The Kolmogorov–Smirnov test of normality and histograms were used to assess the statistical normality assumption of metric variables. Levene's test of homogeneity of variance was used to assess the statistical homogeneity of variance assumption. The chi-square test of independence was used to explore the associations between the categorical variables, while an adjusted likelihood ratio-chi-squared test was used when the expected count assumption of the chi-squared test was violated. An independent samples t-test was used to assess the mean differences of continuous variables across the levels of categorically binary measured variables.

A multivariate binary logistic regression analysis was conducted to assess the combined and individual associations between relevant predictors of exposure of the HCWs to recent physical violence at the workplace. Associations between the measured predictor variables and their outcomes are expressed as odds ratios (ORs) with 95% confidence intervals (CIs). A *p*-value below 0.05 was considered statistically significant.

### Ethical approval

This study was conducted according to the guidelines of the Declaration of Helsinki. Approval was obtained from the institutional review board of King Saud University College of Medicine (approval number: E-18–3391) before the study was started. Written informed consent for participation, publication, and confidentiality was obtained from the study participants at the beginning of the survey.

## Results

### Demographic characteristics

A total of 304,002 HCWs were identified from the SCFHS database; only 7,398 (male, 51.3%; female, 48.7%) responded to the questionnaire. The participants’ mean age was 40 ± 8.62 years, and 60% were of non-Saudi origin. Of the participants, nurses, midwives, and healthcare specialists accounted for 38.1%, physicians for 30.91%, healthcare technicians and ambulance technicians for 25.54%, and pharmacists for 5.43%. Most of the participants were employed full-time (89.86%) in the public/government sector (72.47%). Their work settings were as follows in ascending order: ambulatory, specialized units, general medicine, emergency, intensive care, technical services, management, operating room, general surgery, psychiatric, and support services (Table 1).

**Table 1:** Descriptive analysis of healthcare workers’ sociodemographic and professional characteristics. *N* = 7398

Characteristics	n (%)
Sex	
Male	3792 (51.3)
Female	3606 (48.7)
Age	
20–29 years	402 (5.4)
30–39 years	3752 (50.7)
40–49 years	2143 (29)
50–59 years	882 (11.9)
≥ 60 years	219 (3)
Nationality	
Saudi	2957 (40)
Non-Saudi	4441 (60)
Clinical role	
Physicians	2287 (40)
Pharmacists	402 (5.4)
Nurses, midwives, and healthcare specialist	2819 (38.1)
Healthcare technicians and ambulance personnel	1890 (25.5)
Rank/ seniority	
Junior	4605 (62.2)
Senior	1876 (25.4)
Consultant	917 (12.4)
Experience years	
1–5 years	851 (11.5)
6–10 years	2334 (31.5)
11–15 years	1905 (25.8)
16–20 years	1025 (13.9)
≥ 21 years	1283 (17.3)
Working sector	
Semi-governmental organization	380 (5.1)
Private sector	1656 (22.4)
Public/ governmental sector	5362 (72.5)
Employment type	
Full-time	7256 (98)
Part-time	78 (1.1)
Temporary/ casual	64 (0.9)

### Experience of sexual workplace violence

Only 3.9% of HCWs had experienced a sexual violence incident at their workplace in the last 12 months, with most of the sexual harassments coming from patients (29.5%) or a staff member (27.6%). Most of the survivors pretended that it had never happened (43.3%) immediately after the act, while 36.2% asked the offender to stop, and 28.4% had taken no action against the offender (Table 2).

**Table 2:** Descriptive analysis of healthcare workers’ perceptions and experience of sexual workplace violence

Variable	Total n (%)
Occurrence of sexual violence in the last 12 months, *n* = 7398	
No	7108 (96.1)
Yes	290 (3.9)
Typical incident of violence in your workplace, *n* = 268	
Yes	205 (76.5)
No	63 (23.5)
The attacked person, *n* = 268	
Patient/client/	79 (29.5)
Staff member	74 (27.6)
Relatives of patient/client	49 (18.3)
Management staff member/supervisor	24 (9)
Other persons	17 (6.3)
External colleague/worker	15 (5.6)
General public	10 (3.7)
Place of incident, *n* = 268	
Inside health institution or facility	245 (91.4)
Other place	13 (4.9)
Outside (on way to work/health visit/home)	8 (3)
At patient’s/client’s home	2 (0.7)
Response to the incident, *n* = 268	
Tried to pretend it never happened	116 (43.3)
Told the offending person to stop	97 (36.2)
Took no action	76 (28.4)
Reported it to a senior staff member	53 (19.8)
Told a colleague	48 (17.9)
Told my friends/family members	26 (9.7)
Transferred to another position elsewhere	16 (6)
Completed an Incident/accident report form	14 (5.2)
Took another action	8 (3)
Sought counselling	8 (3)
Sought help from the medical association	4 (1.5)
Completed a compensation claim	1 (0.4)
Sought help from the Saudi commission for healthcare workers	1 (0.4)
Preventability of incident, *n* = 268	
Yes	174 (64.9)
No	94 (35.1)

### Consequences of sexual violence

As shown in Table 3, participants reported a moderate level of disturbance due to their distressing memories, with a self-rated score of 3.4 out of 5 bothering points. Additionally, the participants reported a relatively high level of hyper-alertness related to their experiences of sexual harassment, with a score of 3.93 out of 5 bothering points. In Table 4, 11.2% of the survivors believed an action was taken to investigate the event further by mainly the managers (86.7%). However, 60% of those whose events were investigated reported that a verbal warning was issued to the offenders. The overall satisfaction with the corrective and investigative actions taken to handle the sexual harassment event were between dissatisfied to slightly satisfied (mean satisfaction = 2.16 out of 5). The primary reasons for not reporting the sexual harassment were fear of the negative consequences, thought of reporting being pointless or useless, and shame.

**Table 3:** Bothered about sexual violence

Variable	Point/5
Bothering about attack	
a- Repeated, disturbing memories, thoughts, or images of the attack, mean (standard deviation, SD) Likert rating	3.40 (1.41)
b- Avoiding thinking about or talking about the attack or avoiding having feelings related to it-mean (SD) Likert rating	3.50 (1.36)
c- Being "super-alert" or watchful and on guard -mean (SD) Likert rating	3.93 (1.33)
d- Feeling like everything you did was an effort -mean (SD) Likert rating	3.54 (1.35)

**Table 4:** Consequences of sexual violence

Variable	Total n (%)
Investigation of the causes of the incident, *n* = 268	
No	209 (78)
Yes	30 (11.2)
Don’t know	29 (10.8)
The perpetrator, *n* = 30	
Management staff member/employer	26 (86.7)
Community	2 (6.7)
Other	3 (10)
Police	3 (10)
Medical association	1 (3.3)
Consequences for the perpetrator, *n* = 30	
Verbal warning issued	18 (60)
Don't know	4 (13.3)
None	4 (13.3)
Other	2 (6.7)
Reported to police	1 (3.3)
Aggressor prosecuted	1 (3.3)
The offer of employer or supervisor, *n* = 117	
Opportunity to speak about/report it	58 (92.1)
Other support	32 (50.8)
Counselling	27 (42.9)
Incident handling satisfaction, *n* = 260 Mean (SD) Likert rating, 1 = V dissatisfied, 5 = V. satisfied,	
Very dissatisfied	130 (50)
Dissatisfied	34 (13.1)
Neutral	48 (18.5)
Satisfied	21 (8.1)
Very satisfied	27 (10.4)
Reason for not reporting the incident, *n* = 260	
I was afraid of negative consequences	120 (46.2)
I thought it was useless	104 (40)
I felt ashamed	77 (29.6)
I did not know who to report to the incident	38 (14.6)
It was not important	37 (14.2)
Other	15 (5.8)
I felt guilty	12 (4.6)

### Experience of sexual attacks and their sociodemographic and professional factors

Female HCWs had a higher rate of harassment than male HCWs. The age of the HCWs was significantly associated with exposure to sexual harassment at the workplace in the last 12 months (*p* < 0.001), as a higher proportion of the survivors were in the 30–39 and 20–29 years age groups than in the > 40 years age group. Non-Saudi HCWs were less sexually harassed in the last 12 months than Saudi HCWs (*p* = 0.003). In addition, physicians comprised the least proportion of the survivors (*p* < 0.001), while nurses comprised the greatest proportion. Furthermore, the consultant HCWs had a lower rate of sexual violence in the last year compared to the seniors and juniors (*p* < 0.001). In addition, the proportion of survivors in the working sector did not correlate significantly with exposure to sexual violence, indicating that the HCWs in different sectors may have a nearly equal rate (Table 5).

**Table 5:** Bivariate analysis of the association between healthcare workers’ experience of sexual workplace violence and sociodemographic/professional factors

	Sexually attacked in your workplace n (%)			
	No = 7108	Yes = 290	test statistic	*p*-value
Sex				
Male	3678 (51.7)	114 (39.3)	χ2 (1) = 17.24	< 0.001
Female	3430 (48.3)	176 (60.7)		
Age				
20–29 years	378 (5.3)	24 (8.3)	χ2 (4) = 72.76	< 0.001
30–39 years	3544 (49.9)	208 (71.7)		
40–49 years	2096 (29.5)	47 (16.2)		
50–59 years	874 (12.3)	8 (2.8)		
≥ 60 years	216 (3)	3 (1)		
Nationality				
Saudi	2817 (39.6)	140 (48.3)	χ2 (1) = 8.68	0.003
Non-Saudi	4291 (60.4)	150 (51.7)		
Clinical Role				
Physicians	2226 (31.3)	61 (21)	χ2 (3) = 24.6	< 0.001
Pharmacists	388 (5.5)	14 (4.8)		
Nurses, Midwives, and Health specialists	2670 (37.6)	149 (51.4)		
Healthcare Technicians and Ambulance staff	1824 (25.7)	66 (22.8)		
Rank/seniority				
Junior	4413 (62.1)	192 (66.2)	χ2 (2) = 11.94	0.003
Senior	1795 (25.3)	81 (27.9)		
Consultant	900 (12.7)	17 (5.9)		
Experience years				
1–5 years	814 (11.5)	37 (12.8)	χ2 (4) = 71.70	< 0.001
6–10 years	2190 (30.8)	144 (49.7)		
11–15 years	1829 (25.7)	76 (26.2)		
16–20 years	1004 (14.1)	21 (7.2)		
> 20 years	1271 (17.9)	12 (4.1)		
Working sector				
Public/ governmental sector	5154 (72.5)	208 (71.7)	χ2 (3) = 1.024	0.599
Private- for profit sector	1586 (22.3)	70 (24.1)		
Other semi-governmental/private organization	368 (5.2)	12 (4.1)		
Employment type				
Full-time	6977 (98.2)	279 (96.2)	χ2 (2) = 4.88	0.087
Part-time	73 (1)	5 (1.7)		
Temporary/casual	58 (0.8)	6 (2.1)		

### Experience of sexual attacks and their working conditions

HCWs who worked in shifts, especially those working the night shift (18:00 to 07:00), were significantly more exposed to sexual violence at the workplace (*p* < 0.050) (Table 6). In addition, direct physical contact with the patients was a significant predictor of sexual violence among the HCWs (*p* = 0.001). Moreover, the sex of the patients with whom the HCWs had been working was not a significant predictor of sexual violence (*p* = 0.155).

**Table 6:** Bivariate analysis of the association between healthcare workers’ experience of sexual attack at the workplace with their working condition factors

Variable	Sexually attacked in your workplace (%), *n* = 7398			
	No = 7108	Yes = 290	test statistic	*p*-value
Work in shifts				
No	3082 (43.3)	99 (34.1)	χ2 (1) = 9.67	0.002
Yes	4026 (56.6)	191 (65.9)		
Working time between 18:00 (6 PM) and 07:00 (7 AM)				
No	2636 (37.1)	85 (29.3)	χ2 (1) = 7.24	0.007
Yes	4472 (62.9)	205 (70.7)		
Interacting with patients/clients				
No	726 (10.2)	21 (7.2)	χ2 (1) = 2.71	0.100
Yes	6382 (89.8)	269 (92.8)		
Routine direct physical contact (washing, turning, lifting) with patients/clients				
No	3275 (46.1)	110 (37.9)	χ2 (2) = 14.15	0.001
Yes	3110 (43.8)	159 (54.8)		
Not Applicable	723 (10.2)	21 (7.2)		
Patients/clients you most frequently work with are (tick all appropriate)				
Newborns	1312 (18.5)	58 (20)	χ2 (1) = 0.44	0.508
Infants	1544 (21.7)	69 (23.8)	χ2 (1) = 0.70	0.402
Children	2628 (37)	123 (42.4)	χ2 (1) = 3.53	0.060
Adolescents	3385 (47.6)	162 (55.9)	χ2 (1) = 7.58	0.006
Adults	5594 (78.7)	245 (84.5)	χ2 (1) = 5.60	0.018
Elderly	4030 (56.7)	196 (67.6)	χ2 (1) = 13.50	< 0.001
Sex of the patients you most frequently work with				
Unspecified/not applicable	723 (10.2)	21 (7.2)	χ2 (3) = 5.25	0.155
Female	557 (7.8)	16 (5.5)		
Male	623 (8.8)	27 (9.3)		
Male and female	5205 (73.2)	226 (77.9)		

### Experience of sexual attacks and characteristics from hospital violence reporting guidelines

Violence-related worry was more common in survivors (mean score, 3.5/5 points using a Likert scale, SD = 1.21) than in non-survivors (mean score, 2.82; SD = 1.33) (*p* < 0.001). Furthermore, HCWs working in institutions with policies and guidelines for dealing with work-related violence had a lower rate of WPV than those in institutions with no such policies and guidelines (*p* = 0.006) (Table 7). Encouragement from work to report WPV of any type was a significant predictor of lower rates of sexual WPV in the last year (*p* < 0.001); HCWs in violence-intolerant work environments had a significantly lower exposure to WPV than those in workplaces without violence intolerance policies. Furthermore, encouragement from managers and employers was a significant predictor of lower exposure to sexual violence among the HCWs (*p* < 0.001).

**Table 7:** Bivariate analysis of the association between healthcare workers’ experience of sexual workplace violence and hospital violence reporting guidelines

**Variable**	**Sexually attacked in your workplace (%), *n* = 7398**			
	**No = 7108**	**Yes = 290**	**test statistic**	***p*-value**
Worried about violence in the current workplace, Mean (standard deviation)	2.82 (1.33)	3.49 (1.21)	t (7396) = 8.44	< 0.001
Presence of procedures for reporting of violence				
No	1993 (28)	103 (35.5)	χ2 (1) = 7.67	0.006
Yes	5155 (72)	187 (64.5)		
Knowing how to use report				
No	751 (14.7)	32 (17.1)	χ2 (1) = 0.85	0.358
Yes	4364 (85.3)	155 (82.9)		
Encouragement to report workplace violence				
No	2588 (36.4)	151 (52.1)	χ2 (1) = 29.30	< 0.001
Yes	4520 (*63.6*)	139 (47.9)		
Person who encourages reporting				
Management staff/employer	3824 (53.8)	108 (37.2)	χ2 (1) = 30.68	< 0.001
Colleagues	1432 (20.1)	52 (17.9)	χ2 (1) = 0.85	0.356
Saudi commission for health specialist	555 (7.8)	15 (5.2)	χ2 (1) = 2.72	0.099
Medical association	174 (2.4)	7 (2.4)	χ2 (1) = 0.001	0.971
My own family/friends	357 (5)	19 (6.6)	χ2 (1) = 1.351	0.245
Other persons	287 (4)	20 (6.9)	χ2 (1) = 5.73	0.017

### Relationship between healthcare workers’ experience (in years) and exposure to sexual violence

**Fig. 1:**
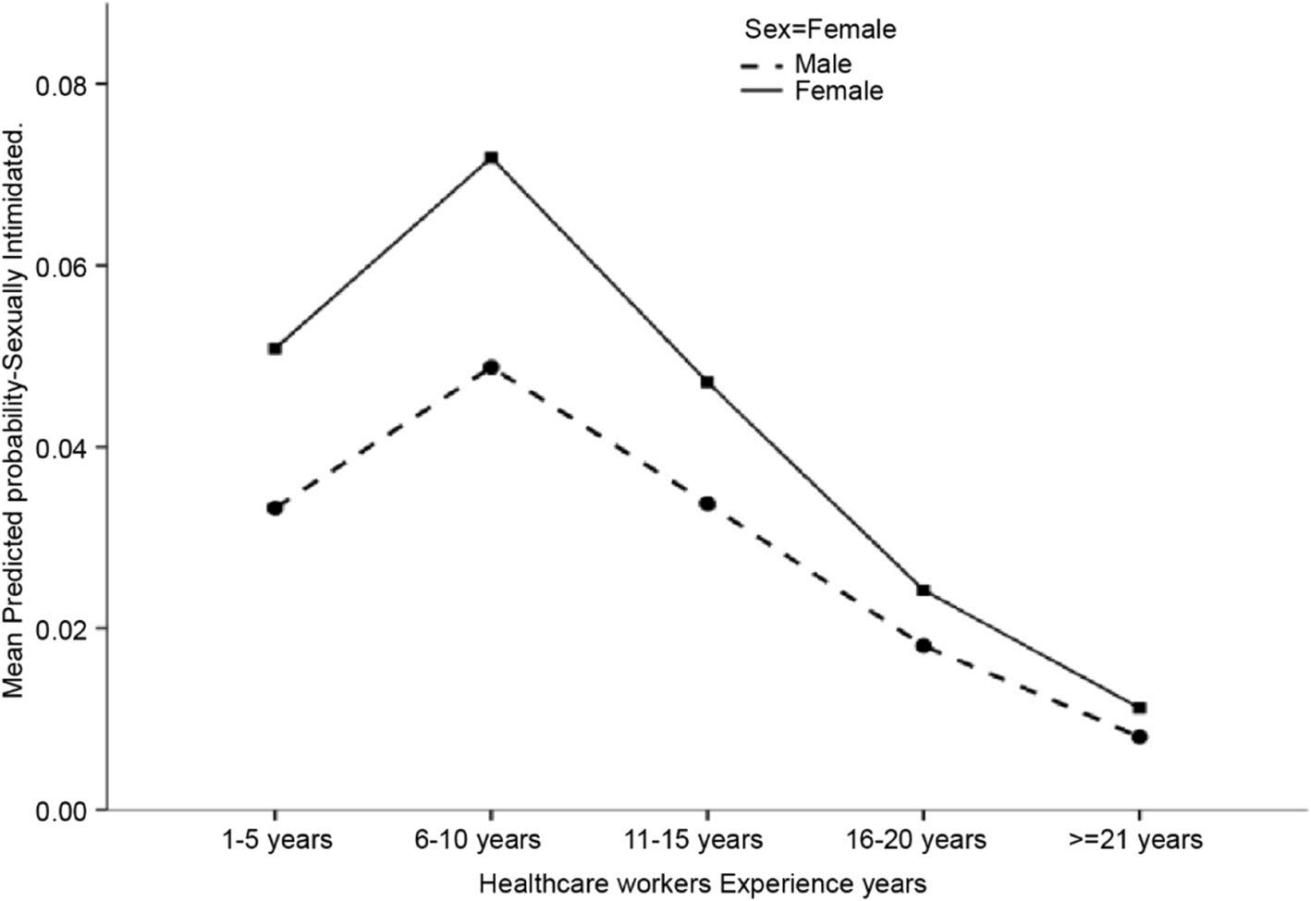
Association between healthcare workers’ experience with risk of sexual workplace violence by sex

Female HCWs had more significant exposure to sexual violence than male HCWs (Fig. 1). HCWs with 6–10 years of experience were the most susceptible group in both sexes. However, a decrease in exposure to sexual violence was observed with an increase in the HCWs’ experience (in years), regardless of sex.

### Multivariate logistic binary regression analysis results

Female HCWs had a more significant exposure to sexual violence in the past year than male HCWs (*p* = 0.002). There was no association between age of HCWs and sexual violence exposure (*p* = 0.227). However, non-Saudi HCWs had a significantly lower (33.3% times less) predicted rate of sexual violence than Saudi HCWs (*p* = 0.005). In addition, nurses had the greatest likelihood of being exposed to sexual WPV (52.8% times higher, *p* = 0.002). There was a significant negative association between HCW’s experience and risk of sexual exposure in the last year. HCWs with 6–10 years of experience were 3.696 times more (269.6% times more) exposed to sexual violence compared to those with ≥ 21 years of experience (*p* < 0.001). People working in the private sector had a greater risk (1.47 times more) of sexual violence than those working in other sectors (*p* = 0.016). The presence of a dedicated guideline/procedure for reporting/handling WPV was not significantly associated with exposure to sexual violence (*p* = 0.649), but encouragement by institution leaders and administrators to report all cases of sexual violence was a significant predictor of a reduced risk of WPV (38.4% times less) (*p* = 0.001). Moreover, HCWs caring for elderly patients during most of their work time were 2.51 times (151% times more) more exposed to sexual WPV than those caring for mainly non-elderly patients (*p* < 0.001) (Table 8).

**Table 8:** Multivariate logistic binary regression analysis of the predictors of exposure to recent sexual workplace violence

	**Multivariate adjusted Odds Ratio (OR)**	**95% C.I. for OR**		***p*-value**
		**Lower**	**Upper**	
Experience, 6–10 years	3.696	1.836	7.442	< 0.001
Experience, 11–15 years	2.668	1.347	5.283	0.005
Encouragement to report from significant others	2.514	1.524	4.147	< 0.001
Experience, 1–5 years	2.413	1.101	5.258	0.028
Experience, 16–20 years	1.797	0.858	3.763	0.120
Sex, Female	1.546	1.180	2.026	0.002
Job, Nurse	1.528	1.171	1.995	0.002
Working sector, Private	1.467	1.073	2.004	0.016
Working with elderly patients	1.390	1.065	1.814	0.016
Worry level from violence at work, mean score	1.340	1.214	1.478	< 0.001
Working shifts, Yes	1.225	0.932	1.610	0.145
Has direct physical contact with clients	1.119	0.904	1.385	0.303
Seniority Level	1.028	0.842	1.254	0.786
Presence of Violence reporting guidelines at workplace	0.936	0.704	1.245	0.649
Age group	0.862	0.678	1.096	0.227
Nationality, Non-Saudi	0.667	0.502	0.885	0.005
Encouragement from institution administration to report	0.616	0.461	0.824	0.001
Constant	0.008			< 0.001

*Abbreviations*: *CI* Confidence interval

*N* = 7398

## Discussion

To the best of our knowledge, this is the first study in Saudi Arabia to estimate the prevalence of sexual WPV in the healthcare sector. A low rate of sexual violence was observed in the present study, as only 3.9% of the participants were exposed. This study showed a significant association between exposure to sexual violence and being a female HCW (*p* = 0.002). The odds of being exposed to sexual violence were 1.5 higher (*p* = 0.002) among nurses. Sexual violence is a significant but not well-documented problem, as no study measured it in all cities in Saudi Arabia according to the authors’ knowledge, which this study discussed.

Most of the studies conducted nationally have mainly focused on a particular city, hospital, or specialty. Al Anazi et al. [18] reported no sexual violence case, possibly because the study was conducted in a small conservative city with a population quite familiar with each other, which has the potential of a negative social impact. The low reporting rates in the study by El-Gilany et al. [19] was due to sex separation in primary healthcare centers. Alharbi et al. [13] reported that almost 75% of their participants experienced sexual violence; this may be due to the different definition of sexual violence used in their study. In addition, most of their participants were female. In a cross-sectional study [11] conducted in Riyadh city among nurses, low sexual violence rates were observed. However, this study was conducted exclusively in Riyadh, and this finding cannot be extrapolated to the entire Saudi population. Moreover, most of the participants were female nurses (78.6%).

Most countries have a high prevalence of sexual violence [3, 20–22]. A cross-sectional study conducted in Iran and involving HCWs reported a sexual violence rate of 4.7% [23], which was consistent with our findings. Like Saudi Arabia, Iran is a conservative community. This could explain the low reporting rate due to the sensitivity of the subject and the lower focus on investigating the prevalence and causes. A systematic review was conducted to estimate the prevalence of sexual violence among native HCWs in high-income countries between 2001 and 2019. The prevalence of sexual violence (both harassment and abuse) among HCWs was 6% [24]. However, those countries are less conservative than second-world countries and have better reporting access and rules to prevent and deal with such events. In contrast, a quantitative review was conducted to estimate the rate of different violence types among nurses worldwide [20]. They had seven-fold higher rates of sexual violence than those in our findings (25%). This might be due to under-reporting of sexual harassment in Saudi Arabia, which means more efforts should be directed toward sexual violence in our region. Another cross-sectional study conducted in Ethiopia reported a higher rate [9]; the authors explained this by the unavailability of a sufficient and well-defined system of identification and control of such incidents, as well as a lack of concern about HCWs’ exposure to sexual violence. We observed that most of the perpetrators were patients/clients and not staff members, which is consistent with other findings [3, 13, 25]. In a study conducted in Macau by Cheung et al. [26], all but four of the survivors were harassed by patients and their relatives; the relatively small number of HCWs in Macau can explain this observation. Contrastingly, Celik et al. [27] found that most perpetrators were staff members. However, they involved only nurses, who answer to physicians, and could have placed them in a vulnerable position due to the power differences. Khoshknab et al. [23] reported that most perpetrators were relatives of patients/clients, followed by patients. Their study was conducted in teaching hospitals in which patients are usually surrounded by students and supervisors, giving the relatives a bigger chance for violence. The physical, psychological, and economical pressures on patients and their family members can account for the several sexual violence cases [28].

Most of the survivors in this study tried to pretend that the incident never happened and some of them told the attacker to stop his/her behavior or took no action. In another study [23], most did nothing or told the person to stop. This implies a very serious outcome—the incident can be repeated. Over three-quarter of the participants said that no investigations were conducted to explore the causes of the incident, which is more than the rates reported by Chen et al. [3] and Khoshknab et al. [23]. Apprehension of possible outcomes and feeling of no ensuing action were reasons for not reporting. A study in India involving women [29] revealed that not reporting may have been due to community standards and beliefs, as responsibility would entirely be shifted to the women’s behavior or attitude or the act would be considered normal. Ignorance about one’s rights may also explain under-reporting, as many are afraid to lose their jobs, especially those in the private sector or with temporary jobs. A study in China [30] reported that when the perpetrator was a co-worker, the survivor was unlikely to report the incident. This could be because the survivor does not want to be stigmatized in the workplace or is afraid of the potential outcomes, especially if the attacker has a higher position. Repeated exposure to sexual violence can make the HCW tolerate the act and consider it normal in their daily work [31]; this can also explain non-reporting. Conversely, nurses in Turkey [27] with a low educational level would rather not report sexual harassment, which may be due to their low knowledge of their rights or because they have a lower working position. Song et al. [32] found lack of knowledge of how and what incidents to report, lesser attention to the healthcare providers compared to the patients, and previous experience of no action taken by the authorities after reporting as the main reasons for not reporting. Most survivors were harassed during their night shifts between 18:00 to 07:00, which contrasts the findings of Khoshknab et al. [23] who reported that most incidents happen during morning shifts. The difference in work hours can explain the discrepancy.

In this study, female workers were more exposed to sexual WPV, which is consistent with some findings [22, 33–35] and not consistent with others [30, 36]. El-Gilany et al. [19] and Wang et al. [25] found no sex differences between survivors, while Alharbi et al. [13] observed a non-significant difference. In contrast to female workers, male workers may normalize sexual violence in the workplace because they perceive some situations as more sexually oriented than their female counterparts [37]; this may account for the low reporting rates among male survivors. Torre et al. [38] also found no sex differences. However, most of the participants were young (20–24 years), indicating less experience on how to act in such an event. Consistent with our findings, most of the survivors in the study by Fujita et al. [22] were nurses, explained by the high need for direct physical contact and interaction. Yenealem et al. [34] reported that almost all survivors had little work experience (1–5 years) and that 75% of them had procedures for reporting violence in their workplaces. This study found that juniors and HCWs with 6–10 years of experience are more vulnerable to sexual violence, implying that little experience could translate to lack of skills to manage such incidents [34]. In another study conducted in Addis Ababa, most of the nurses who were survivors were single and young, implying their lack of experience in handling such situations and their reverence for higher healthcare providers in their society [39].

The consequences of sexual violence include psychological stress, shame, depression, sleep disturbances, impaired practice, and unhealthy and uneasy relationships with patients [28, 36, 40]. Moreover, the survivor may refer several patients to other colleagues and ask for unnecessary investigations to get rid of the aggressor, which may subsequently lead to a greater cost [27]. Another study conducted in Iran [41] found that some survivors lost their jobs because of absenteeism following violence-related trauma. Some survivors even quit their jobs or prevented their children from working in their field or having a relationship with someone of the same profession. Moreover, some survivors’ relationship with their spouse was negatively affected, as their spouse would blame them for what happened or starting being more suspicious. This may contribute to under-reporting in future and family divisions. Zeighami et al. [42] conducted a qualitative study and proposed some strategies to prevent sexual violence by interviewing nurses with a prior experience. They found that portraying a strict attitude with the perpetrator such as being inactive or behaving ignorantly of the bad behavior, having a professional relationship and not talking or making jokes on private matters, and wearing an unattractive uniform so not to tempt others would stop him/her from continuing. In addition, having the healthcare provider care for the same sex or having a staff member with more experience in the same shift, providing more protective measures for HCWs on night shifts, and changing the workplace for HCWs with a prior experience, are good preventive measures. Nonetheless, education and training on sexual violence should be provided early in schools, colleges, and workplaces. Further, having a zero-tolerance policy by taking immediate legal actions should be promoted. Longitudinal studies are needed to explore the reasons for sexual violence and implement solutions accordingly. More awareness through educational programs and the media for HCWs, patients, and their relatives is important. In addition, a more encouraging environment to report every violent incident with strict consequences for the attackers should be implemented. More importantly, new regulations (e.g., more staff members, shorter waiting times, and more support, such as prevention programs) are necessary.

### Limitations

The main limitation of this study is its use of a retrospective self-report questionnaire, which might cause recall bias. In addition, the subject’s sensitive nature may have prevented some workers from participating, resulting in low reporting rates and reporting bias. In addition, although this study had a sufficiently large number of participants, the results cannot be generalized to the entire population.

The strength of this study is that the participants were all HCWs from government or private institutions in Saudi Arabia, unlike previous studies that focused mainly on the emergency departments and nurses in specific cities.

## Conclusion

The prevalence of sexual violence is low; however, it remains a risk faced by HCWs, especially those working night shifts and having direct physical contact with patients. The prevalence was highest among nurses, midwives, and healthcare specialists and lowest among physicians. To explore the causes of sexual violence and to implement solutions accordingly, further studies, especially longitudinal, are needed. Educational programs for HCWs, patients, and their relatives are required. Furthermore, increasing awareness using the media is important. The underreporting of cases may skew the magnitude of the problem; thus, a more encouraging environment to report every violence incident with strict consequences for the perpetrators should be implemented. More importantly, new regulations (e.g., more staff members, shorter waiting time, and more support such as prevention programs) are necessary.

## Data Availability

The datasets used and/or analyzed during the current study are available from the corresponding author on reasonable request.

## Abbreviations

CI: Confidence interval
HCW: Healthcare worker
OR: Odds ratio
SCFHS: Saudi Commission for Health Specialty
SD: Standard deviation
WHO: World Health Organization
WPV: Workplace violence
